# Low Molecular Weight Hyaluronan-Pulsed Human Dendritic Cells Showed Increased Migration Capacity and Induced Resistance to Tumor Chemoattraction

**DOI:** 10.1371/journal.pone.0107944

**Published:** 2014-09-19

**Authors:** Manglio Rizzo, Juan Bayo, Flavia Piccioni, Mariana Malvicini, Esteban Fiore, Estanislao Peixoto, Mariana G. García, Jorge B. Aquino, Ariel Gonzalez Campaña, Gustavo Podestá, Marcelo Terres, Oscar Andriani, Laura Alaniz, Guillermo Mazzolini

**Affiliations:** 1 Gene Therapy Laboratory, Facultad de Ciencias Biomédicas, Universidad Austral, Derqui-Pilar, Buenos Aires, Argentina; 2 CONICET (Consejo Nacional de Investigaciones Científicas y Técnicas), CABA, Buenos Aires, Argentina; 3 Department of Surgery, Hospital Austral, Universidad Austral, Derqui-Pilar, Buenos Aires, Argentina; 4 CIT NOBA, Universidad Nacional del Noroeste de la Pcia de Bs. As (UNNOBA), Junín, Buenos Aires, Argentina; University of Lyon, France

## Abstract

We have shown that *ex vivo* pre-conditioning of bone marrow-derived dendritic cells (DC) with low molecular weight hyaluronan (LMW HA) induces antitumor immunity against colorectal carcinoma (CRC) in mice. In the present study we investigated the effects of LMW HA priming on human-tumor-pulsed monocytes-derived dendritic cells (DC/TL) obtained from healthy donors and patients with CRC. LMW HA treatment resulted in an improved maturation state of DC/TL and an enhanced mixed leucocyte reaction activity *in vivo*. Importantly, pre-conditioning of DC/TL with LMW HA increased their ability to migrate and reduced their attraction to human tumor derived supernatants. These effects were associated with increased CCR7 expression levels in DC. Indeed, a significant increase in migratory response toward CCL21 was observed in LMW HA primed tumor-pulsed monocyte-derived dendritic cells (DC/TL/LMW HA) when compared to LWM HA untreated cells (DC/TL). Moreover, LMW HA priming modulated other mechanisms implicated in DC migration toward lymph nodes such as the metalloproteinase activity. Furthermore, it also resulted in a significant reduction in DC migratory capacity toward tumor supernatant and IL8 *in vitro*. Consistently, LMW HA dramatically enhanced *in vivo* DC recruitment to tumor-regional lymph nodes and reduced DC migration toward tumor tissue. This study shows that LMW HA –a poorly immunogenic molecule- represents a promising candidate to improve human DC maturation protocols in the context of DC-based vaccines development, due to its ability to enhance their immunogenic properties as well as their migratory capacity toward lymph nodes instead of tumors.

## Introduction

Colorectal carcinoma (CRC) is a one of the leading causes of cancer-related death worldwide. Unfortunately, there is no curative treatment for patients who are not amenable of surgical resection [1]. Thus, new therapeutic strategies are needed for advanced CRC patients and those based on mounting immune responses against tumors might play a key role [2], [3].

Dendritic cells (DC) are professional antigen presenting cells that have the capacity to generate innate and adaptive immune responses, and are essential to induce immunity against cancer [4]. DC migrate from peripheral blood to different organs and tissues wherein they capture antigens and process them to form MHC-II-peptide complexes. This non-activated (immature) DC can present self-antigens to T cells, which leads to immune tolerance either through T cell deletion or through the differentiation of regulatory or suppressor T cells [5]. By contrast, activated (mature) antigen-loaded DC, which express some specific molecules such as CD40, CD80 and CD86, can launch the differentiation of antigen-specific T cells into effect tor T cells with unique functions and cytokine pro­files [6]. The use of mature DC to prime responses to tumor associated antigens (TAA) provides a promising approach for cancer immunotherapy, but clinically relevant responses have been rather poor until now [7].

Issues regarding the optimal dose and route of administration for DC vaccination used in cancer therapy remain to be addressed. In fact, only a small proportion of DC intradermally injected reaches the draining lymph nodes [8], [9]. We have previously demonstrated both *in vivo* and *in vitro* that DC pre-conditioning with low molecular weight hyaluronan (LMW HA) is able to enhance DC migration toward regional lymph nodes in mice [10]. This effect was shown to be independent of two of the HA receptors, CD44 and TLR4, and to be likely mediated, at least partially, by an increased CCR7 expression [11]. CCR7 is a key molecule which interacts with the chemokines CCL19 and CCL21 and it was found to be crucial for guiding DC migration from peripheral tissues to draining lymph nodes [12]–[14].

A number of cytokines and factors have been used as culture medium supplement in order to increase such migratory capacity in DC, including PGE2 and the TLR3 agonist Poly (I:C) [15]. However, these compounds might also induce the expression of IDO (indoleamine-2,3-dioxygenase) and thereby would eventually suppress immune responses or generate tolerogenic DC [15], [16]. In addition, it has been observed that poly (I:C) and LPS can affect the maturation process of peripheral blood monocytes by inducing the so-called suppressors of cytokine signaling (SOCS) activation [17], [18].

Tumors induce immunosuppression by secreting different cytokines and immunosuppressive molecules which finally interfere with approaches aimed at generating anti-cancer immunity [19]. Among them, IL-8 is a chemokine produced in large amounts by the majority of tumors [20]. IL-8 has been implicated in the resistance to antiangiogenic therapies [21] and in the failure of DC-based immunotherapy protocols [22]. In fact, DC can produce and respond to IL-8, a molecule shown to inhibit anti-tumor immune responses when it was peri/intra-tumoraly injected [23].

Hyaluronic acid (HA) is a lineal, large and ubiquitous glycosaminoglycan with a simple chemical structure found mainly in tissues undergoing cell proliferation, regeneration and repair [24]. HA functions are well known to be size-dependent and the LMW HA form has been shown to induce the expression of pro-inflammatory genes such as IL-8, IL-12, TNF-α and inducible NO synthase in many types of cells including DC [25], [26]. In addition, LMW HA or its small fragments were shown to stimulate T cell responses by activating and up-regulating co-stimulatory molecules on DC in a CD44-independent and TLR4-dependent manner [27], [28]. Moreover, it has also been demonstrated that LMW HA can act as an adjuvant promoting antigen-specific T cell responses *in vivo* through TLR2 stimulation [29].

The aim of this work was to evaluate the effects of LMW HA pre-conditioning on tumor-pulsed human DC obtained from both healthy donors (HD) and CRC patients. We found that LMW HA induced DC/TL maturation state able to enhance lymphocyte proliferation, and most importantly increasing their migratory capacity and avoiding tumor tissue attraction. These results provide the rationale for the potential use of LMW HA as an immunostimulatory molecule for DC-based vaccines protocols in the treatment of cancer patients.

## Materials and Methods

### Reagents

Pharmaceutical endotoxin-free LMW HA of definite size (1–3×10^5 ^Da) from CPN spol.s.r.o (Czech Republic) was supplied by Farmatrade (Buenos Aires, Argentina). A stock solution of 5 mg/ml LMW HA was prepared, and the presence of endotoxins was determined by Limulus amebocyte lysate (LAL) assays with a sensitivity limit of 0.05–0.1 endotoxin units (EU) per ml (Sigma–Aldrich). GM-CSF was obtained from PrepoTech or Bioprofarma; CCL21, from PeproTech, and IL-4 and Poly (I:C), from Invitrogen.

### Blood and tumor tissue

CRC patients and HD were recruited in the study after signing informed consent forms. Peripheral blood sample and colorectal carcinoma tissue were obtained at the time of surgical resection at the Austral University Hospital. Informed consent was obtained from all patients in accordance with our IRB.

### Generation of dendritic cells

Peripheral blood mononuclear cells (PBMC) were isolated by Ficoll-Paque gradient. Cells were plated into 6-well plates for 2 h. Then, adhered cells were subsequently cultured for up to 7 days in RPMI 1640 medium (Invitrogen), containing 10% fetal calf serum (Invitrogen), 2 mM L-glutamine, 100 U/mL penicillin, 100 µg/ml streptomycin, human recombinant GM-CSF (Bioprofarma, Growgen, 350 ng/ml), human recombinant IL-4 (IL-4 R&D Systems 500 UI/ml) and 2-βMercaptoethanol (0.05 M). In HA stimulation experiments, cells were treated from day 3 with LMW HA (50 µg/ml). At day 7, DC were pulsed with autologous tumor lysates (200 µg/10^6^ cells/ml) for 12–18 h, with or without LMW HA (50 µg/ml). Poly (I:C) (10 µg/ml) treatment was used for DC activation and as another control condition. Cells were then centrifuged and used for experiments.

### Tumor lysates

Tumor tissues were frozen at −80°C until use. Then, tumor samples were dispersed with needle and scalpel and disrupted by 5 freeze-thaw cycles. For large debris removal, tumor lysates were centrifuged at 300 rpm for 10 min. The supernatant was collected and passed through a 0.22 µm Millipore Express, sterile, low binding proteins filter unit. The protein concentration of the lysate was determined by Bradford assay. The resulting tumor lysates were aliquoted and stored at −80°C until use.

### Flow cytometry

Staining and flow cytometric analyses of generated DC were carried out using standard procedures. Briefly, cells were stained with different conjugated antibodies as follows: anti-CD11c (B-ly6), anti-MHC-II (G46-6), anti-CD40 (5C3), anti-CD80 (L307.4), anti-CD86 (2331), and their respective isotypes controls (BD Biosciences, San Diego, CA, USA) on ice for 30 min, washed thoroughly with PBS-1% BSA. Cells were then fixed with 1% paraformaldehyde and subjected to flow cytometry (FACSCalibur, BectonDickinson-BD, USA). Data were analyzed using Cyflogic software.

### Mixed lymphocyte reaction

Tumor-free female *Nu/Nu* nude mice were intraperitoneally injected with 1.5×10^6^ DC/TL pre-treated or not with LMW HA, and with allogeneic healthy human-derived CFSE-labeled PBLs (5×10^6^). Forty eight hours later, cells were obtained by intraperitoneal lavages and incubated with mouse anti-human CD3 antibody for 30 min. They were subsequently incubated with biotinylated anti-mouse antibody and avidin-Texas Red for 45 min. Samples were analyzed using a FACSCalibur Flow Cytometer (Becton Dickinson). For FACS analysis lymphocytes were gated based on CD3^+^ marks and the number of T cell divisions was measured as proportional to the dilution of CFSE intensity.

### Lymph node and tumor conditioned medium generation

Tumoral tissues were obtained from *Nu/Nu* nude mice carrying human s.c. implanted CRC fresh fragments. Mice were sacrificed; lymph node and tumor tissue were removed and minced into pieces smaller than 1 mm^3^. To obtain lymph node (LNCM) and tumor conditioned medium (TCM), the minced fragments were transferred into a 24 well tissue culture plate (6 fragments/well) with 500 µL of DMEM (plus 2 µmol/L glutamine, 100 U/mL penicillin and 100 mg/mL streptomycin) with 0.05% BSA but without FBS. The supernatant was collected 24 h later and stored at –80°C until use.

### *In vitro* migration assays

For *in vitro* migration assays, micro-chemotaxis Boyden Chamber units were used. DC/TL (1,5–2,5×10^4^) treated or not with LMW HA (50 µg/ml) or Poly (I:C) (10 µg/ml) were placed into the upper chamber of the transwell unit. In some assays DC/TL treated or not with LMW HA were preincubated for 24 h with an IL-8 neutralizing antibody (20 µg/ml). Chemoattractant medium containing 100 ng/ml of CCL21, LNCM, TCM, human recombinant IL-8 (20 ng/ml) or DMEM as negative control was placed in the lower chamber of the transwell unit. In blocking assays neutralizing IL-8 or isotype antibody (20 µg/ml) was added to TCM. The system was incubated for 4 h at 37°C in a 5% CO_2_ humidified atmosphere. Cells which migrated through the membrane pores were stained with DAPI and counted using UV microscopy with a 10x objective lens: 5 fields per well were analyzed and the mean number of cells/field ± SEM were calculated. Results are shown as DC/TL and DC/TL/LMW HA migration index. Poly (I:C) was used as a positive control maturation stimulus.

### Zymography

Metalloproteinase activity was determined by zymography. Supernatant of culture from DC/TL treated or not with LMW HA was run on a 10% SDS PAGE containing 0.1% gelatin (Sigma-Aldrich). The gel was stained with Coomassie Brilliant Blue R-250 for 30 min at room temperature. Gelatinase activity was visualized by negative staining; gel images were obtained with a digital camera (Canon EOS 5D), and were subjected to densitometric analysis using Scion Image software (Scion Corporation, Frederick, MD). Relative MMP-2 activity was obtained by normalizing values to untreated samples (DC/TL). HT1080 cell line supernatant was used as positive control of MMP-2 and MMP-9 activity [30].

### qPCR

Total RNA from DC/TL treated or not with LMW HA was extracted by Tri Reagent (Sigma-Aldrich Co., St. Louis MO, USA). Two micrograms of RNA were reverse transcribed with 200 U of SuperScript II Reverse Transcriptase (Invitrogen, Carlsbad, CA, USA) and 500 ng of Oligo (dT) primers. cDNAs were subjected to real time PCR. Each 25 µl reaction volume contained 1 unit Taq DNA polymerase (Invitrogen), 1× PCR reaction buffer (20 mM Tris-HCl, pH 8.4, and 50 mM KCl), 1.5 mM Mg_2_Cl, 200 µM of dNTPs and 0.4 µM of each specific primer: TLR2 forward 5′-GGGTTGAAGCACTGGACAAT-3′ and reverse 5′-CTGCCCTTGCAGATACCATT-3′; TLR4 forward 5′-TGAGCAGTCGTGCTGGTATC-3′ and reverse 5′-CAGGGCTTTTCTGAGTCGTC-3′; CD44 forward 5′-GCGCAGATCGATTTGAATTAA-3′ and reverse 5′-GTGCCCTTCTATGAACCCAT-3′; IL-8 forward 5′-GGTGCAGTTTTGCCAAGGAG-3′ and reverse 5′-TTCCTTGGGGTCCAGACAGA-3′; CXCR1 forward 5′-TTTTCCGCCAGGCTTACCAT-3′ and reverse 5′- AACACCATCCGCCATTTTGC-3′; CXCR2 forward 5′- TAAGTGGAGCCCCGTGGGG-3′ and reverse 5′- TGGGCTCAGGGGCAGGATG-3′. PCR conditions were: 90 seconds at 94°C and then 40 cycles of 30 seconds at 94°C, 30 seconds at 60°C and 30 seconds at 72°C. Values were normalized to levels of glyceraldehyde-3-phosphate dehydrogenase (GAPDH; used as housekeeping) transcript (forward 5′-CATCTCTGCCCCCTCTGCTG-3′ and reverse 5′-GCCTGCTTCACCACCTTCTTG-3′). cDNA was quantified using the OligoGreen Single Stranded Quantification kit (Invitrogen) according to the manufacturer’s instructions. Data were processed by the ΔΔCt method. The relative amount of the PCR product amplified from untreated cells was set as 1. A non-template control (NTC) was run in every assay, and all determinations were performed as triplicates in three separated experiments.

### Tumor tissue cytokine array

Tumor conditioned medium was obtained as previously described and detection of human chemokines was performed using Human Chemokine Antibody Array C1 (RayBio C-Series) according to the manufacturer's instructions. The chemokines analyzed were: BLC, CCL28, CCL23, CTACK, CXCL16, EN78, Eotaxin1, Eotaxin2, Eotaxin3, Fractalkine, GCP2, GRO, GROα, HCC4, I-309, I-TAC, IL-8, IP-10, XCL1, MCP-1, MCP-2, MCP-3, MCP-4, MDC, MIG, MIP-1α, MIP-1β, MIP-1δ, MIP-3α, MIP-3β, MPIF1, NAP2, PARC, RANTES, SDF-1α, SDF-1β, TARC, TECK.

### *In vivo* migration assays

DC/TL treated or not with LMW HA and stained with CM-Dil were inoculated subcutaneously into the back of nude mice, between tumor and nearest lymph node, after 72 h of s.c. tumor implantation. At 48 h mice were sacrificed and tumor and ipsilateral inguinal lymph nodes were removed, dissected and dissociated by enzymatic digestion with D-collagenase (Cabiochem). Red blood cells were removed using a lysis solution (0.15 M NH_4_Cl, 1 mM KHCO_3_, 0.1 Na_2_-EDTA). The remnant cells were subsequently fixed in 1% paraformaldehyde and subjected to flow cytometric analysis (FACSCalibur, Becton–Dickinson, BD). Data were processed using Cyflogic software.

### Ethics statement

Human cells and tumor tissues were obtained from healthy donors/patients after written informed consent and protocol were approved by the “Institutional Evaluation Committee” (CIE) from School of Biomedical Sciences, Austral University (Protocol No. 12-019). Animals were maintained at our Animal Resource Facilities (School of Biomedical Sciences, Austral University) in accordance with the experimental ethical committee and the NIH guidelines on the ethical use of animals. The “Animal Care Committee” from School of Biomedical Sciences, Austral University, approved the experimental protocol. All surgery was performed under isoflurane anesthesia, and all efforts were made to minimize suffering.

### Statistical analysis

Paired t test or Mann–Whitney (InStat, GraphPad Software) were used for statistical analyses. Differences with p values ≤0.05 were considered as statistically significant.

## Results

### Patient characteristics and dendritic cells generation

Fifteen HD and 25 CRC patients were recruited in this study. Nineteen patients had liver metastasis and 39% had received previous chemotherapy. DC were generated from PBMC. DC were co-cultured with autologous tumor lysates (TL) at day 6 for 18 h. In HA condition, LMW HA (50 µg/ml) was added to the culture medium (see Materials and Methods). At day 6, cultured DC displayed a relatively immature phenotype but became more mature when they were pulsed with autologous TL. When CD11^+^ DC/TL were gated we evidenced that LMW HA pre-incubation induced a statistically significant up-regulation of MHC-II and CD86 expression in HD DC (p≤0.05) (table 1).

**Table 1:** Phenotipic analyses of LMW HA pre-treated DC.

	Healthy donors (HD)				CRC Patients			
	DC	DC/TL	DC/TL/LMW HA	DC/TL/Poly I:C	DC	DC/TL	DC/TL/LMW HA	DC/TL/Poly I:C
**MHC II**	220	224δ	238*	217	135	174	192	180
**CD86**	265	225δ	238*	218	109	171	170	148
**CD83**	164	157	160	157	101	93	112	133
**CD40**	120	113	108	123	397	384	375	381

PBMC-derived DC from healthy donors (HD) and colorectal carcinoma (CRC) patients were stained with mAbs anti-CD11c, MHC-II, CD86, CD83 and CD40. CD11c^+^ cells were gated and the co-expression of several markers was analyzed. Data are expressed as geometric mean fluorescence.

*vs HD DC/LT,

δ vs CRC patients DC/TL;

p≤0,05.

### Dendritic cells are potent stimulators in mixed lymphocyte reaction after LMW HA stimulation

**Figure 1:**
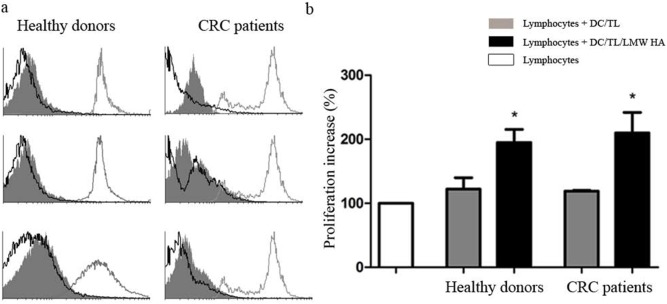
LMW HA treatment of DC enhances human T-cell proliferation *in vivo.* Nude mice received intraperitoneal injections of human-derived CFSE-labeled PBLs (5×10^6^) and allogeneic mature DC/TL, or DC/TL/LMW HA (1,5×10^6^). Proliferation was monitored 48 h later by FACS-gated lymphocytes from peritoneal lavages by quantifying fluorescent dye signal which is inversely correlated to their proliferation rate. Fluorescence intensity in the input undivided lymphocytes was over 95% (gray line in the histogram). a) Three histograms representative of both HD and CRC patients are shown. Gray line: undivided lymphocytes; gray shadow: Lymphocytes + DC/TL; black line: Lymphocytes + DC/TL/LMW HA. b) Bars represent percentage of lymphocyte proliferation increase ± SEM respect to undivided lymphocytes (HD n: 5 and CRC patients n: 5). White bar: lymphocyte alone; gray bar: DC/TL; black bar: DC/TL/LMW HA. *DC/TL vs DC/TL/LMW HA; Paired t test; p≤0.05.

We then asked whether LMW HA treatment would impact on DC antigen presentation. To this end we performed an *in vivo* MLR assay using allogeneic CFSE-labeled PBL from HD and LMW HA primed/non-primed DC/TL which were injected inside nude mice peritoneal cavity as previously described [23]. As a result, a 72% and 80% increase in PBL proliferation activity was observed in the LMW HA primed DC/TL (DC/TL/LMW HA) condition when compared to LMW HA non-primed DC/TL (DC/TL) one in HD or CRC patients, respectively (p≤0,05) (Fig. 1, b).

### LMW HA treatment increases migratory response of DC toward CCL21 and induces their CCR7 expression

**Figure 2:**
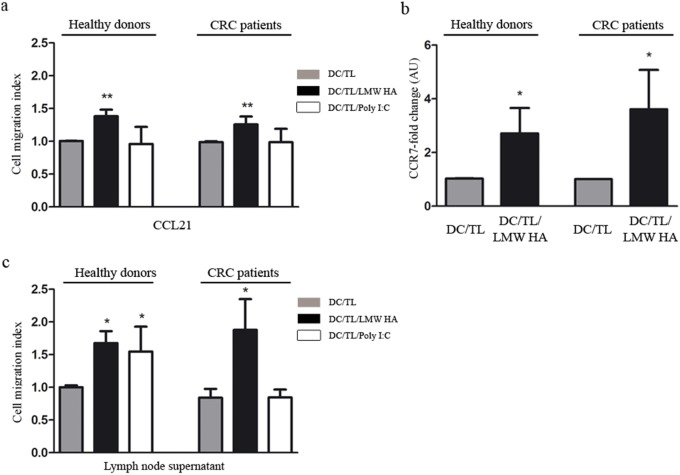
LMW HA treatment increases migratory response of DC toward CCL21 inducing their CCR7 expression and increases their migratory response toward lymph node supernatant. a) Migratory ability of DC treated or not with LMW HA toward CCR7 ligand CCL21 (200 ng/ml) was evaluated in a Boyden chamber system. DC were loaded into the upper well of the chamber and CCL21 was added to the lower chamber. Data are expressed as DC/TL and DC/TL/LMW HA migration index. Poly (I:C) was used as a positive control of maturation stimulus. b) CCR7 expression was examined by qPCR. Black bars represent CCR7 fold-change expression in comparison with DC/TL. c) Similar chemotaxis assays were set up placing lymph node supernatant from tumor bearing nude mice in the lower chamber as chemoattractant. Bars represent DC migration index from both HD and CRC patients. Gray bar: DC/TL; black bar: DC/TL/LMW HA; white bar: DC/TL/Poly (I:C). DC/TL vs DC/TL/LMW HA. Mann Whitney t test; * p≤0.05; **p≤0.01.

The process of DC migration is insufficiently understood [31]. Once DC capture tumor antigens, DC acquire the ability to cross through the endothelium and to migrate through the extracellular matrix and thereby entering the lymph node inner cortex [32]–[34]. Indeed, it is well known that both lymph nodes and tumors secrete several cytokines which might attract DC [22]. To further understand mechanisms involved in LMW HA effects on DC we analyzed the CCL21/CCR7 axis and found an increased ability to migrate toward CCL21 in DC/TL/LMW HA *in vitro* when compared to DC/TL condition, in both HD or CRC patients (38% and 25%, respectively; p≤0,01) (Fig. 2, a). In addition, treatment with LMW HA upregulated the expression levels of CCR7 in DC (HD DC fold change 2,7; and CRC patients DC fold change 3,6; p≤0,05) (Fig. 2, b).

### LMW HA treatment increase DC migratory capacity toward lymph node supernatant

Migration of DC to lymph node allows their interaction with naïve T cells, a critical step in the induction of immunity against cancer and a challenge for DC-based vaccine protocols [10]. Therefore, we decided to mimic *in vitro* the conditions faced by DC when they are inoculated *in vivo* by using lymph node conditioned medium (LNCM) as a chemoattractant. Interestingly, a potent migration of DC toward LNCM was found when they were pre-incubated with LMW HA in comparison with DC/TL condition. In contrast, poly I:C induced migration of DC only when they derive from HD (Fig. 2, c).

### LMW HA priming of DC reduces their migratory response to IL-8 and inhibited their attraction to tumor conditioned medium

**Figure 3:**
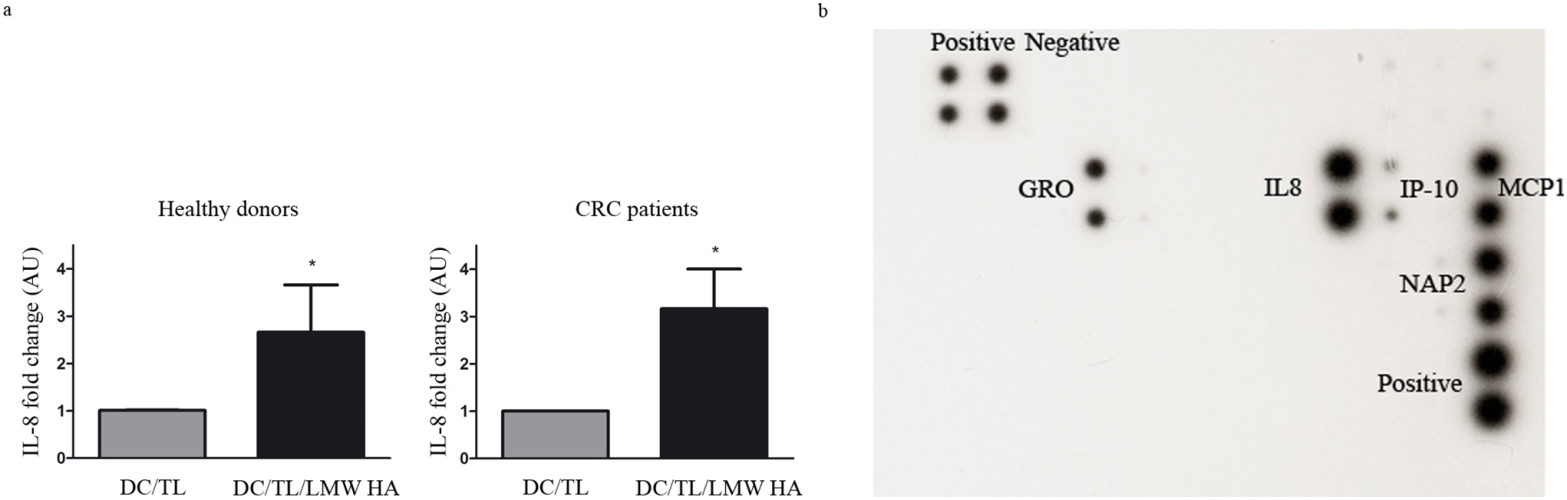
IL-8 expression is upregulated in LMW HA treated DC and is present in tumor supernatant. a) IL-8 expression was evaluated by qPCR. b) Human chemokine antibody array (38 proteins) was performed from tumor culture supernatant. The signal intensity for each antigen-specific antibody spot is proportional to the relative concentration of the antigen in the sample. Gray bars: DC/LT; black bars: DC/TL/LMW HA. Mann Whitney t test; *p≤0,05. * vs. DC/TL.

**Figure 4:**
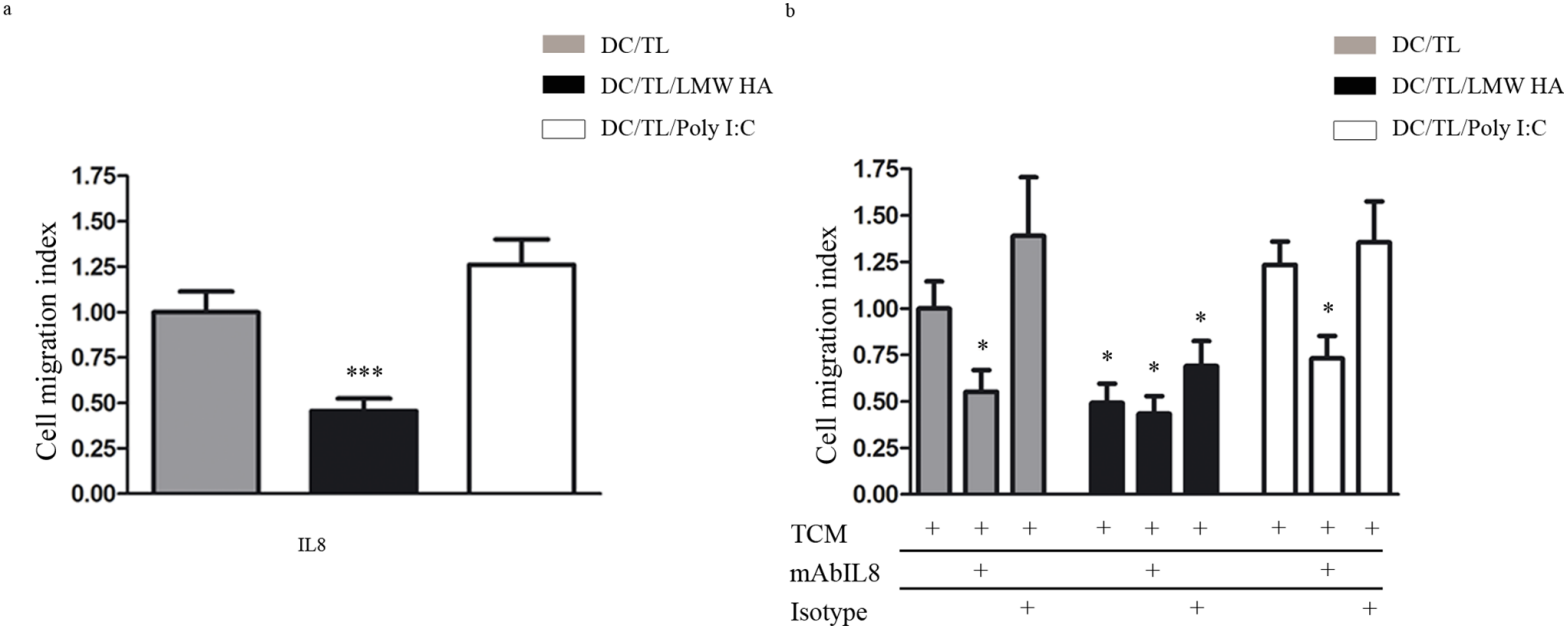
LMW HA induces resistance in human DC to IL-8 chemoattraction. a) Chemotaxis assays were set up placing IL-8 in the lower chamber and LMW HA treated or untreated DC were placed in the upper chamber. b) A similar migration assay was set up placing TCM with addition of a specific antibody against IL-8 (mAbIL-8) or isotype antibody (20 µg/ml) in the bottom chamber as chemoattractant. Graphs represent data from 4 independent experiments similarly performed expressed as migration index respect to DC/TL migration toward TCM; five fields were counted for each quadruplicate well. Gray bars: DC/LT; black bars: DC/TL/LMW HA; white bars: DC/TL/Poly (I:C). Mann Whitney t test; *p≤0,05; ***p≤0,001. * vs. DC/TL.

It was recently described that DC IL-8 pre-exposure abrogated DC tumor attraction [23]. We found that LMW HA treatment up-regulated IL-8 expression in DC/TL (Fig. 3, a). Thus, to study whether LMW HA pre-conditioned DC might decrease their response to tumor-derived signals we decided to analyze the IL-8/CXCR axis. After the confirmation of IL-8 expression in tumor conditioned medium (TCM) by a cytokine array assay performed to explore changes in the profile of chemokines secreted by tumors (Fig. 3, b) we observed that only LMW HA pre-treatment decreased by 50% the fraction of DC that migrated toward human recombinant IL-8 (Fig. 4, a). It is worth noting that a reduce migration of DC towards TCM was only observed in DC/TL/LMW HA experimental group when compared to DC/TL control (Fig. 4, b). Moreover, when IL-8 was blocked with an anti-IL-8 mAb a 40% reduction in DC migratory response to TCM was observed in DC/TL or DC/TL/Poly (I:C) conditions, while no changes were observed on DC/TL/LMW HA group (Fig. 4, b). These *in vitro* results suggest the possibility that IL-8 is important for DC attraction toward tumors and that LMW HA pre-treatment may ameliorate this effect.

### Pre-treatment with LMW HA induces metalloproteinase activity and modulates the expression of HA receptors in DC

**Figure 5:**
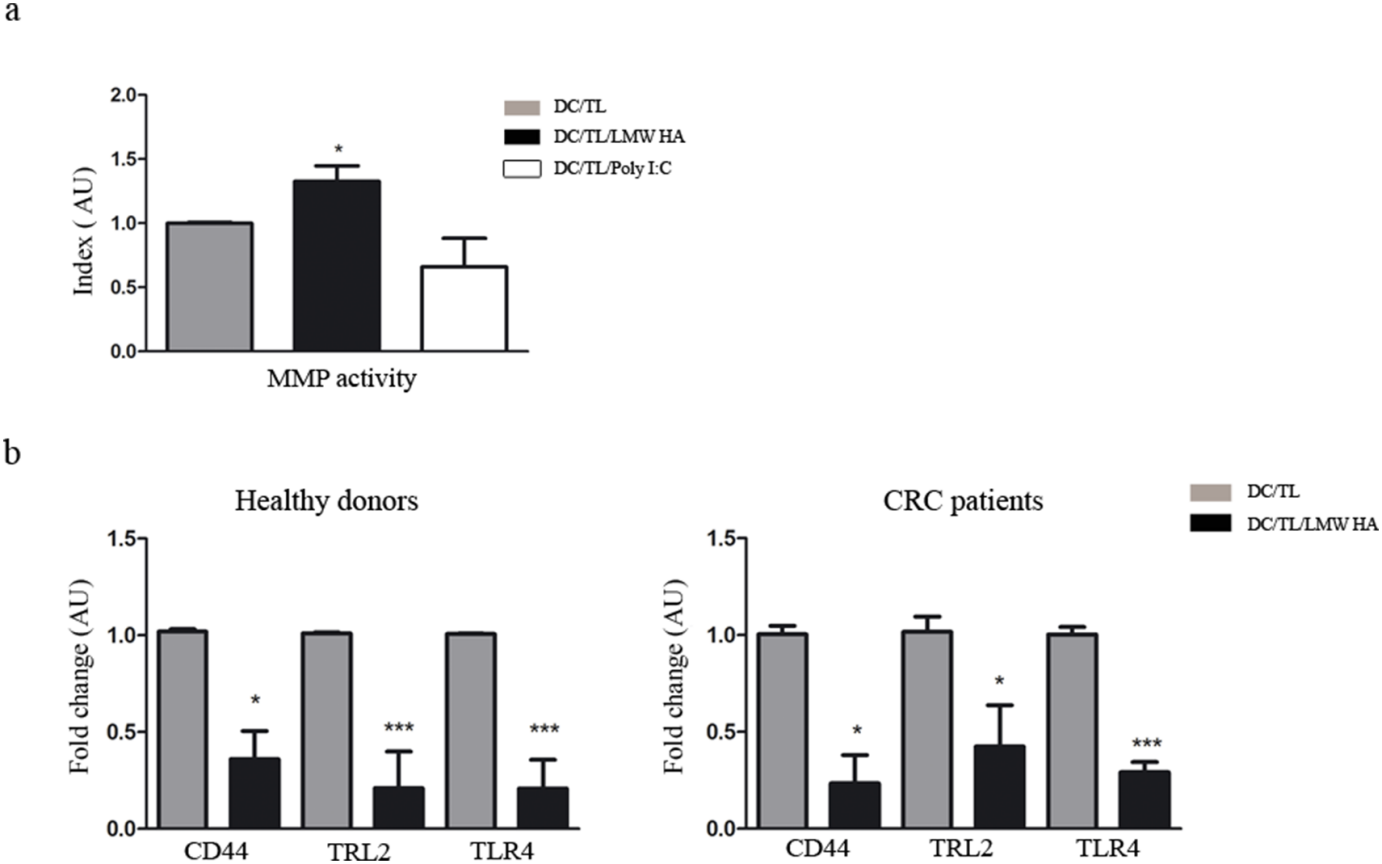
DC pre-treated with LMW HA induces MMP activity and downregulates HA receptors expression. a) MMP activity was analyzed by zymography and quantified by densitometry. Relative MMP activity was obtained by normalizing values to untreated samples (DC/TL). Bars represent MMP activity from HD. b) qPCR of HA receptors was performed. The results are expressed as fold change related to DC/TL. Gray bar: DC/TL; black bar: DC/TL/LMW HA. DC/TL vs DC/TL/LMW HA. Mann Whitney t test; *p≤0,05; ***p≤0,001.

Considering that metalloproteinases (MMPs) participate in DC migration through the ECM [35] we further evaluated if LMW HA might affect DC/TL MMP’s activity by zymography. Indeed, pre-incubation of DC with LMW HA resulted in a 30% increase in the gelatinolytic activity when compared to control (Fig. 5, a). HA is a component of the ECM with the capacity to induce cell signaling mechanisms on cells through several receptors such as CD44 [36], TLR-2 [29], and TLR-4 [37]. These receptors have been implicated in DC maturation as well in adhesion and migration processes. To further study the effects of LMW HA on DC/TL we analyzed its impact on HA receptors expression by qRT-PCR. Interestingly, a down-regulation of the HA receptors CD44, TLR-2, and TLR-4 was found in DC/TL/LMW HA derived from HD and CRC patients when compared to DC/TL control group (Fig. 5, b).

### DC pre-incubated with LMW HA show increased *in vivo* migration capacity towards lymph node

**Figure 6:**
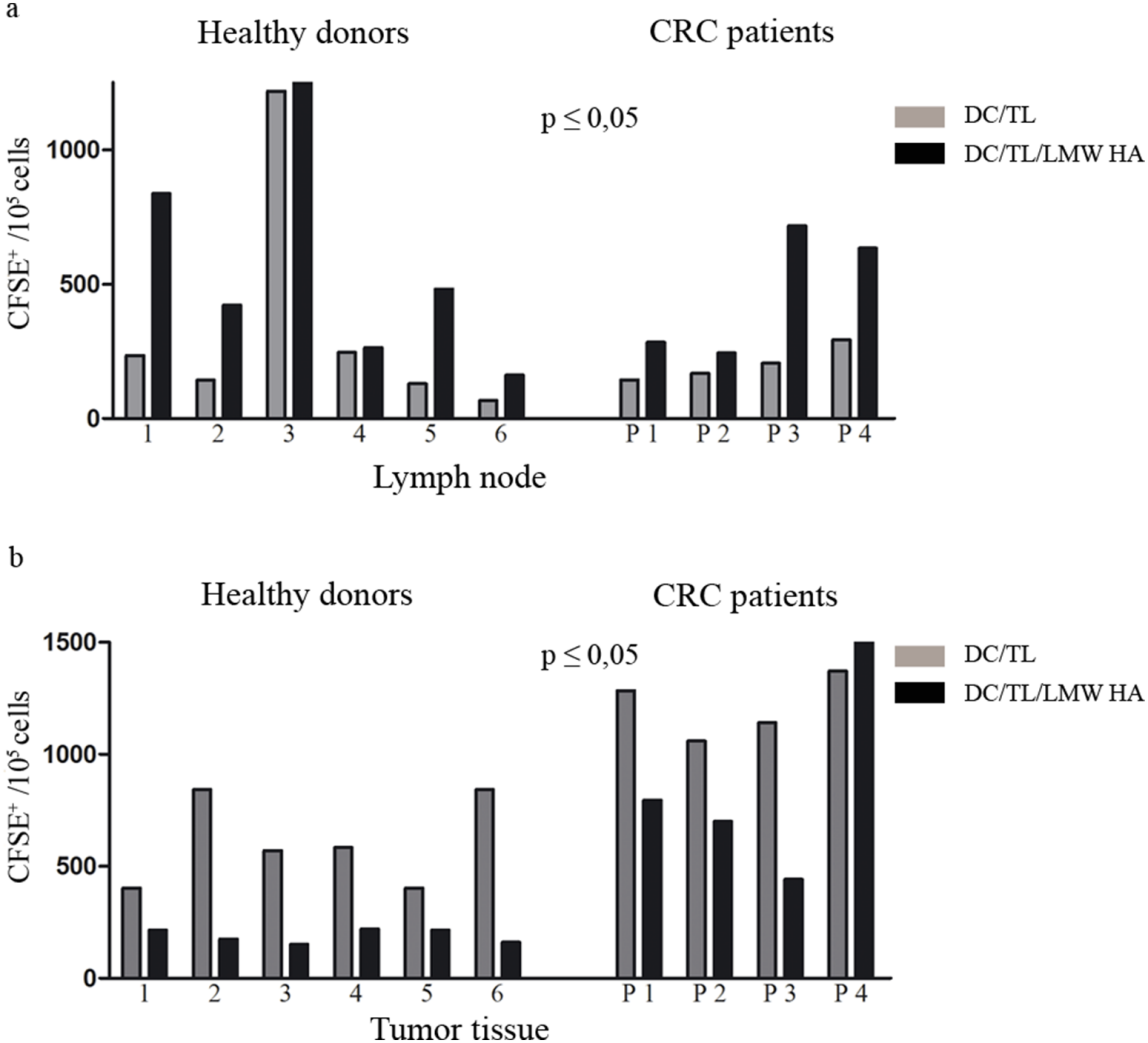
Pre-incubation of DC with LMW HA increases their migration toward lymph node and reduces their tumor attraction *in vivo.* DC/TL were or not LMW HA treated, and after CM DiL labeling were inoculated s.c. in human-tumor bearing nude mice. Twenty four hours later lymph nodes (a) and tumors (b) were surgically removed and a cell suspension was obtained from these tissues. Number of fluorescent DC was counted by flow cytometry. The upper panel (a) shows DC/TL migration toward lymph nodes. The lower panel shows migration toward tumor tissue (b). Bars represent the number of migrated DC per individual HD (1–6) and CRC patients (P 1–4). Gray bar: DC/TL; black bar: DC/TL/LMW HA. * DC/TL vs DC/TL/LMW HA. Paired t test; *p≤0,05.

We have previously reported that bone marrow-derived DC/TL pre-incubated with LMW HA show a higher capability to migrate toward regional lymph node once they were inoculated subcutaneously in a murine model of CRC [11]. Based on that, we aimed at analyzing this feature in PBMC-derived human DC obtained from HD and CRC patients. For this purpose, we chose a nude mouse xenograft model based on generating tumors from human CRC samples. DC/TL treated or not with LMW HA and stained with CMDil were subcutaneously inoculated in mice in a region between the tumor and regional lymph node. As shown in Fig. 6 and when compared to DC/TL, DC/TL/LMW HA from HD and CRC patients migrated more efficiently toward regional lymph nodes but were less attracted to tumors (lymph node: HD 460±253 vs. 703±229 cells, CRC 202±32 vs. 469±120 cells; tumor: HD 608±81 vs. 193±12 cells; CRC 1162±65 vs. 646±105 cells; DC/TL vs. DC/TL/LMW HA, respectively).

## Discussion

A number of strategies for cancer immunotherapy are currently under preclinical and clinical evaluation [38]. Activation and stimulation of DC migration are key steps for the induction of a potent and specific antitumor T cell response. To date, several DC-based cancer vaccination strategies have been employed in the clinic with limited success [39]–[41]; although in 2010 the FDA approved the first antigen presenting cell therapeutic cancer vaccine to prolong survival of patients with advanced hormone-refractory prostate cancer [42]. Different “cocktails” containing cytokines, growth factors and other compounds have been used for maturation and activation of DC including IL-1β, IL-6, TNF, IFN-α, IFN-γ, PGE2 and Poly(I:C) [43], [44]. Our previous results showed that pre-incubation of DC with LMW HA was able to induce an efficient antitumoral effect in a CRC mouse model which was found to be mediated by the stimulation of DC maturation and activation, as well as by the induction of a potent migratory capacity towards lymphoid areas *in vitro* and *in vivo* [11]. In the present study we demonstrated that LMW HA represents a potent stimulator of migration toward lymph node for human DC obtained from both HD and CRC patients, likely partially involving CCR7/CCL21 axis and inducing resistance to IL-8, a tumor-derived DC chemoattractant. In addition, LMW HA treatment enhanced MLR-stimulating capacity of DC in an *in vivo* assay. Thus, this work adds new data regarding the ability of LMW HA to improve migratory capacity of DC that could be relevant in future design of potential cancer vaccines protocols.

Different studies showed that oligosaccharides of HA, but not high molecular weight (HMW) HA, can be used to stimulate immune responses involving DC activation [27], [29], [45], [46]. Nevertheless, this is the first report addressing LMW HA effects on DC derived from patients with cancer and on their migratory response to lymph node signals. As expected, DC/TL from HD showed higher expression levels of maturation markers than those obtained from CRC patients. LMW HA was able to induce stimulatory effects on DC promoting an up-regulation of MHC-II and the co-stimulatory molecule CD86 when obtained from HD samples but not in those from CRC patients. This result could be, at least in part, due to the immunosuppressive state of cancer patients [47], [48]. However, both groups of DC have the capacity to induce alloreactivity of human lymphocytes in a MLR model. In addition, LMW HA treatment was found to induce a down-regulation of TLR-2 and TLR-4 in both HD volunteers and CRC patients. These receptors belong to a large family of membrane proteins with cytoplasmic signaling domains and extracellular domains capable of recognizing pathogen-associated molecular patterns (PAMPs). Segal *et al.* have followed the expression of these molecules during the differentiation process of monocytes to activated dendritic cells. Down-regulation of these receptors might suggest a mature DC phenotype as it was demonstrated in LPS stimulated DC [49]. Therefore, it seems that LMW HA has a stimulatory effect on DC pulsed with tumor lysate in both HD and CRC patients.

Clinical application of DC-based cancer vaccines has been limited due to their weak efficacy. One limitation might be related to the failure of DC to reach secondary lymphoid organs. It is known that only a small percentage of inoculated DC migrate toward the lymph nodes where they could activate naïve T cells [8], [50], [51]. Once DC are inoculated they undergo a kind of conflicting force attraction between tumoral- and lymphoid-derived chemokines. This contrary stimulus and the specific repertoire receptors expressed by DC determine whether they would migrate to the lymph node (for antigenic presentation and lymphocyte activation) or to tumors. It has been shown that different DC maturation stimuli such as IL1β, TNF-α, PGE_2_ or poly (I:C) induce the expression of CCR7 which enhances DC migration response toward lymph node. However, those molecules might exert a negative effect on DC in different ways [52]. For example, it was shown that PGE_2_ and Poly (I:C) can induce a tolerogenic state on DC [16]. Moreover, some studies reported that LPS and poly (I:C) can induce inhibitory effects on DC by activation SOCS signals [17], [18]. We have herein shown *in vitro* evidences that LMW HA pre-conditioning enhances DC migration to CCL21 and inhibits their attraction toward IL-8. Despite it is likely that many molecules involved in DC migration do not cross-react across species differences, we demonstrated *in vitro* and *in vivo* that DC/TL/LMW HA increase their migration toward LNCM from tumor bearing mice and lymph node, respectively. In addition their attraction to TCM and tumor tissue was also diminished. These phenomena seem to be at least partially mediated by upregulation of CCR7 expression levels, which is the main receptor of CCL19 and CCL21. This cytokine is expressed by lymphatic endothelial and lymph node stromal cells, and was shown essential to attract DC toward them [12]–[14].

Another mechanism that might affect DC recruitment toward the lymph node is the capacity to digest extracellular matrix by MMPs, thus facilitating migration through connective tissues and crossing basement membranes [35]. MMPs also act cleaving cell surface receptors or their ligands, promoting migration by releasing or activating ligands for receptors that control cell motility, or suppressing migration by inactivating chemokines [53]. In line with this, Ratzinger *et al*. demonstrated that MMP-2 and MMP-9 are involved in the emigration of DC from epidermal skin explants as well as dermal DC from the dermis [35]. In addition, it was previously shown in DC derived from healthy volunteers [27] as well as in macrophages [54] and in tumor cell lines [55], that HA-oligosaccharide but not HMW HA up-regulates the activity of these MMPs through TLR-4 activation and by promoting CD44-EGFR interaction enhancing Akt signaling and cell migration events [56]. We herein showed that LMW HA was able to induce MMP activity in DC derived from healthy volunteers.

It is well established that HA size is critical for HA function. In fact, only HA fragments induce inflammatory cytokines in different cell types [57]. For this reason we used LMW HA of defined size (1−3×10^5 ^Da) in our experiments. LMW HA can act through several known receptors such as CD44, TLR2 and TLR4 which are expressed on immune cells such as monocytes and DC [37], [58]. CD44 is the major cell surface receptor for HA and has an important role in cell adhesion and migration [59]. We found that LMW HA treatment inhibited the expression of this receptor in DC/TL. Nevertheless, CD44 down-regulation not only did not impair cell migration but also might instead help DC detachment from ECM and migration through interstitial tissue. Indeed, we have found that bone marrow derived DC from both wild type and CD44-deficient mice have a comparable capacity to migrate to CCL21 *in vitro* [11]. Moreover, it was also shown that CD44 deficient mice exhibited an improvement of epidermal LC migration to LNs after epicutaneous sensitization with protein antigen and, additionally, they showed stronger predominant Th responses after immunization [60].

Another key finding of this study was that LMW HA induced the expression of IL-8 in DC/TL. Enhanced IL-8 expression in response to HA fragments has previously been described in several cellular types such as endothelial [37], epithelial [61] and cancer cells [62]. IL-8 is a pro-inflammatory cytokine present at large amount in cancer microenvironment and, noteworthy, involved in cell migration process. In fact, transcription of both IL-8 and MMP-2 has been reported to be regulated through NFκβ [63] which in turn is activated by HA [62]. Luca *et al*. evidenced that IL-8 affects DC migratory capacity by increasing MMP-2 expression [64]. In addition, IL-8 acts in an autocrine and paracrine fashion on melanoma cells causing their enhanced migratory capacity [65]. Furthermore, it was described that pre-exposure to IL-8 results in desensitization of the DC to the chemotactic effects of IL-8. This effect is relevant taking into account the large production of this molecule by tumor cell and their critical role in DC intratumoral retention [22]. It is important to emphasize that IL-8 was found not being able to modify neither DC maturation nor CXCR1 and CXCR2 expression [22], [23]. Altogether, these effects could explain, at least in part, both the higher DC migration into LNs and the lower attraction to tumor tissue without impairment in their immunologic status.

Finally, this study showed for the first time that LMW HA -a highly conserved and poorly immunogenic molecule- represents a promising candidate to be used in DC maturation protocols in order to increase their activation status as well as to improve their migratory capacity toward lymph nodes and inhibited their recruitment into tumors.
